# A Sterol from Soft Coral Induces Apoptosis and Autophagy in MCF-7 Breast Cancer Cells

**DOI:** 10.3390/md16070238

**Published:** 2018-07-17

**Authors:** Jing-Ru Weng, Chang-Fang Chiu, Jing-Lan Hu, Chia-Hsien Feng, Chiung-Yao Huang, Li-Yuan Bai, Jyh-Horng Sheu

**Affiliations:** 1 Department of Marine Biotechnology and Resources, National Sun Yat-sen University, Kaohsiung 80424, Taiwan; jrweng@mail.nsysu.edu.tw (J.-R.W.); m055020016@student.nsysu.edu.tw (J.-L.H.); huangcy@mail.nsysu.edu.tw (C.-Y.H.); 2 Department of Medical Research, China Medical University Hospital, Taichung 40447, Taiwan; 3 Division of Hematology and Oncology, Department of Internal Medicine, China Medical University Hospital, Taichung 40402, Taiwan; d5686@mail.cmuh.org.tw; 4 Cancer Center, China Medical University Hospital, Taichung 40447, Taiwan; 5 Department of Fragrance and Cosmetic Science, College of Pharmacy, Kaohsiung Medical University, Kaohsiung 80708, Taiwan; chfeng@cc.kmu.edu.tw; 6 College of Medicine, China Medical University, Taichung 40402, Taiwan; 7 Graduate Institute of Natural Products, Kaohsiung Medical University, Kaohsiung 80708, Taiwan; 8 Frontier Center for Ocean Science and Technology, National Sun Yat-sen University, Kaohsiung 80424, Taiwan

**Keywords:** PPARγ, sterol, apoptosis, autophagy, breast cancer

## Abstract

The peroxisome proliferator-activated receptor γ (PPARγ) is a nuclear receptor that plays a key role in regulating cellular metabolism, and is a therapeutic target for cancer therapy. To search for potential PPARγ activators, a compound library comprising 11 marine compounds was examined. Among them, a sterol, 3β,11-dihydroxy-9,11-secogorgost-5-en-9-one (compound **1**), showed the highest PPARγ activity with an IC_50_ value of 8.3 μM for inhibiting human breast adenocarcinoma cell (MCF-7) growth. Western blotting experiments showed that compound **1** induces caspase activation and PARP cleavage. In addition, compound **1** modulated the expression of various PPARγ-regulated downstream biomarkers including cyclin D1, cyclin-dependent kinase (CDK)6, B-cell lymphoma 2 (Bcl-2), p38, and extracellular-signal-regulated kinase (ERK). Moreover, compound **1** increased reactive oxygen species (ROS) generation, upregulated the phosphorylation and expression of H2AX, and induced autophagy. Interestingly, pre-treatment with the autophagy inhibitor 3-methyladenine rescued cells from compound **1**-induced growth inhibition, which indicates that the cytotoxic effect of compound **1** is, in part, attributable to its ability to induce autophagy. In conclusion, these findings suggest the translational potential of compound **1** in breast cancer therapy.

## 1. Introduction

It has been reported that energy dysregulation leads to chronic diseases including type II diabetes, cardiovascular diseases, and malignancies [1]. According to the Framingham Offspring cohort study, there is a 17% incidence of obesity-related cancers, including breast, prostate, and colorectal cancers [2]. With the increasing prevalence of metabolic syndromes and cancer, strategies to break the links are urgently needed.

Peroxisome proliferator-activated receptors (PPARs) belong to a subfamily of the nuclear superfamily of ligand-inducible transcription factors, which primarily control the expression of gene networks involved in adipogenesis, lipid metabolism, inflammation, and the maintenance of metabolic homeostasis [3]. There are three subtypes of PPARs: PPARα, PPARβ/δ, and PPARγ, each encoded by different genes and with varying tissue expression and ligand selectivities [4]. Originally, thiazolidinediones (TZDs), synthetic PPARγ activators, have been used for the treatment of patients with type II diabetes through modulating PPARγ-targeted genes including cyclin D1, inflammatory cytokines, and NF-κB [5,6]. Although the mechanism underlying the anti-tumor effects of TZDs remains unclear, multiple studies have shown that TZDs induce apoptosis in many types of human cancer cells, including those of breast, lung, and retinoblastoma [6,7,8]. In addition, several lines of evidence have suggested that the anti-tumor effects of TZDs are independent of PPARγ activation [9,10]. These PPARγ-independent signaling pathways have been reported to contribute to TZDs-induced apoptosis, including those mediated by ER stress [11], c-Myc [12], and ubiquitin-dependent proteasomal degradation of cell cycle- and apoptosis-regulatory proteins such as β–catenin and Sp1 [10]. However, clinical use of TZDs has been associated with severe side effects, including hepatotoxicity, edema, bone fractures, and heart enlargement, which eventually led to the withdrawal of TZDs from the market [13,14]. Interestingly, no similar side effects were reported for the endogenous PPARγ activators including prostaglandin J2 (PGJ2) and fatty acids [3].

Nature products offer a promising compound library of drug screening. To date, many natural compounds have been identified as PPARγ activators, including resveratrol, quercetin, curcumin, and kaempferol [15,16,17]. It has been reported that resveratrol inhibited cell growth and invasion in head and neck squamous cell carcinoma cells [17], and that curcumin inhibited angiogenesis in hepatic stellate cells through PPARγ activation [18]. Previously, we have reported the antitumor or anti-inflammatory activities of a number of marine natural products [19,20]. In this study, we examined the effect of these compounds on PPARγ activation, which led to the identification of compound **1** (3β,11-dihydroxy-9,11-secogorgost-5-en-9-one) as a PPARγ activator. The mechanism by which compound **1** facilitates its anti-tumor activity was also investigated.

## 2. Results and Discussion

### 2.1. Screening for PPARγ Activators from a Small Marine Compound Library

**Figure 1:**
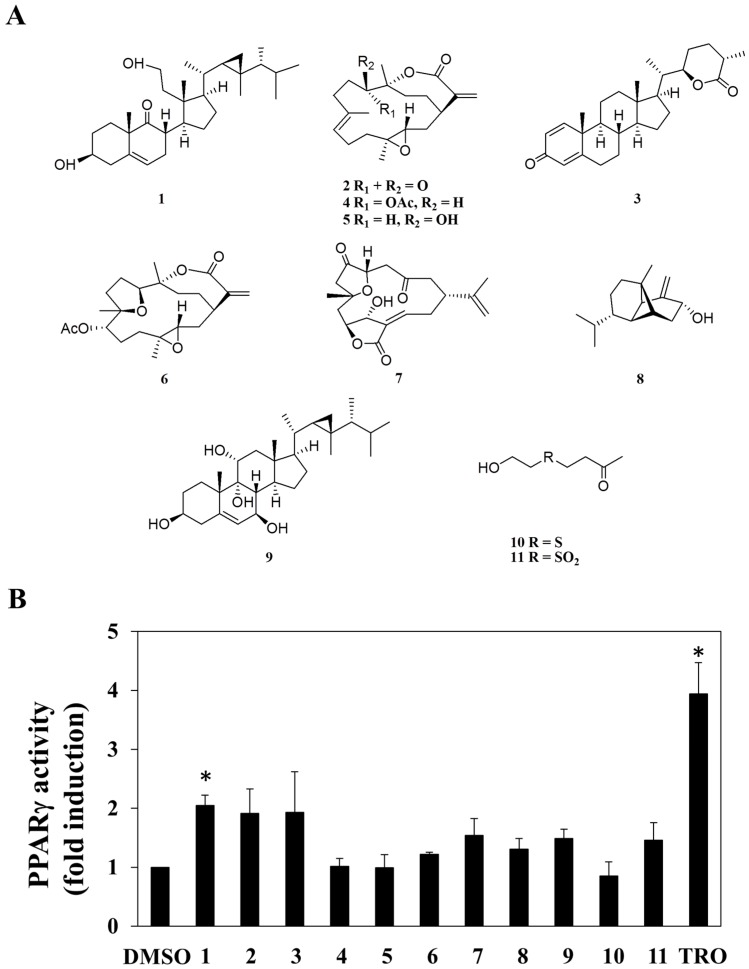
Structures of compounds **1**–**11** (**A**), and the fold change induced by the 11 individual compounds at a dose of 30 μM in peroxisome proliferator-activated receptor γ (PPARγ) promoter region transactivation in human breast adenocarcinoma cell (MCF-7) cells [50 μM troglitazone (TRO) was used as the positive control] (**B**). Each data point represents the mean ± S.D. (*n* = 3). * *p* < 0.05.

A small library consisting of 11 purified marine natural compounds and derivatives from previous studies [19,20,21,22,23,24,25,26,27], was used as a screening platform. These compounds include 3β,11-dihydroxy-9,11-secogorgost-5-en-9-one (**1**) [19], 11-dehydrosinulariolide (**2**) [20], minabeolide-4 (**3**) [21], 11-*epi*-sinulariolide acetate (**4**) [22], sinulariolide (**5**) [20], 3,4;8,11-bisepoxy-7-acetoxycembra-15(17)-en-1,12-olide (**6**) [23], 5-*epi*-sinuleptolide (**7**) [24], lemnalol (**8**) [25], klyflaccisteroid I (**9**) [26], 4-(2-hydroxy-ethylsulfanyl)-butan-2-one (**10**), and dihydroaustrasulfone alcohol (**11**) [27] (Figure 1A).

The ability of these 11 compounds to activate PPARγ in human adenocarcinoma (MCF-7) breast cancer cells was assessed by using an established peroxisome proliferator-activated receptor response element (PPRE)-luciferase reporter assay [28]. Among these compounds, compound **1** exhibited a significant increase in *PPARγ* promoter transactivation in MCF-7 cells (Figure 1B; troglitazone as a positive control), while no PPARγ-activating activity was found in other compounds tested. Pursuant to this finding, we interrogated the role of PPARγ in mediating the anti-proliferative effect of compound **1** in MCF-7 cells.

### 2.2. Compound ***1*** Inhibits Cell Growth in Part through PPARγ Activation

**Figure 2:**
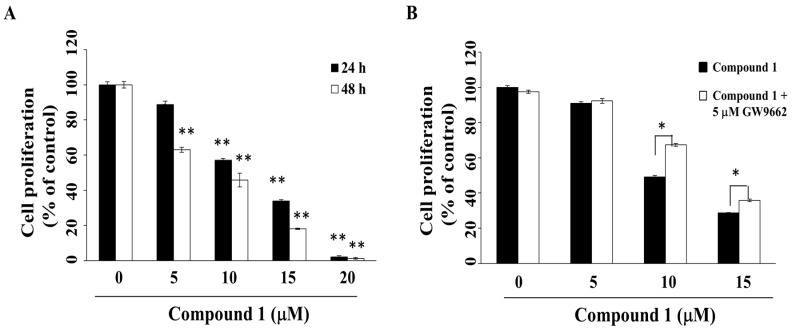
Suppressive effects of compound **1** on the cell proliferation of MCF-7 breast cancer cells. (**A**) Cells were treated with compound **1** at indicated concentrations for 24 h or 48 h, and cell proliferation was determined by MTT assays. Value, mean ± S.D. (*n* = 4–6). ** *p* < 0.01 compared to control; (**B**) Cell proliferation of MCF-7 cells treated with compound **1** at indicated concentrations versus vehicle control for 48 h with or without co-treatment with 5 μM GW9662. * *p* < 0.05.

Previous studies have demonstrated the ability of PPARγ activators to induce cell cycle arrest, differentiation, and apoptosis in many types of cancer cells, including those of pancreatic cancer, hepatoma, and cervical cancer [29,30,31,32]. The anti-proliferative effect of compound **1** was investigated by using 3-(4,5-dimethylthiazol-2-yl)-2,5-diphenyl-2*H*-tetrazolium bromide (MTT) assays. As shown in Figure 2A, compound **1** suppressed the viability of MCF-7 cells in a concentration- and time-dependent manner, with IC_50_ values of 12.2 μM and 8.3 μM at 24 h and 48 h, respectively. Compound **1** was also effective in suppressing the viability of another breast cancer cell line, MDA-MB-231 (Figure S1), indicating that this antiproliferative effect was not cell line-specific.

To evaluate the putative role of PPARγ activation in mediating the anti-proliferative activity of compound **1**, MCF-7 cells were with compound **1** in the presence of the PPARγ inhibitor, GW9662 [33]. As shown in Figure 2B, GW9662 was able to partially protect cells from compound **1**-induced cytotoxicity (*p* < 0.05).

### 2.3. Compound ***1*** Induces Caspase-Dependent Apoptosis

**Figure 3:**
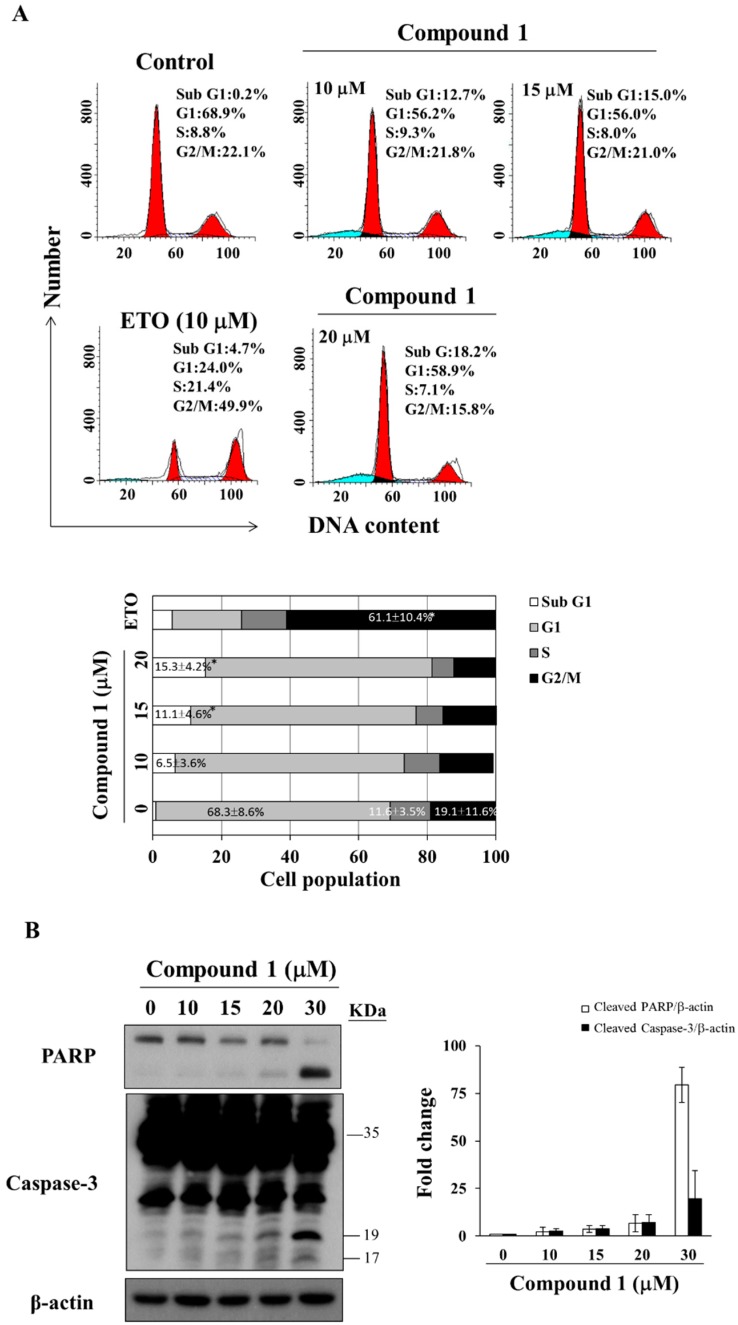
Effect of compound **1** on cell cycle distribution. (**A**) The upper panel shows MCF-7 cells treated with compound **1** at indicated concentrations for 48 h, followed by propidium iodide (PI) staining and flow cytometric analysis. The blue color means cells in sub G1 phase, left side red peak—cells in G1 phase, and right side red peak—cells in G2/M phase. Etoposide (ETO; 10 µM) was used as a positive control. The lower panel shows the average of the three independent experiments. Values, mean ± S.D. (*n* = 3). * *p* < 0.05 compared to control; (**B**) Left panel: effects of compound **1** at indicated concentrations on the expression of PARP and caspase-3 in MCF-7 cells after 48 h of treatment. Right panel: fold changes of cleaved PARP/β-actin and cleaved caspase-3/β-actin in compound **1**-treated MCF-7 cells compared with DMSO control (*n* = 3).

To investigate the mode of antiproliferative action of compound **1**, we examined its effect on the cell cycle distribution of MCF-7 cells via propidium iodide (PI) staining. Flow cytometry analysis revealed that compound **1** caused sub G1 accumulation in a dose-dependent manner after 48 h of treatment (Figure 3A, etoposide as positive control [34]). Compared to the control group, compound **1** increased the population of sub G1 cells from 0.9 ± 1.2% to 15.3 ± 4.2% at 20 μM (Figure 3A). For MDA-MB-231 cells, compound **1** caused G1 accumulation at concentrations below 15 μM (Figure S2). Furthermore, Western blot analysis demonstrated that compound **1** increased PARP cleavage and caspase-3 activation in a dose-dependent manner in MCF-7 cells (Figure 3B).

### 2.4. Compound ***1*** Upregulates the Expression of PPARγ Target Gene Products

**Figure 4:**
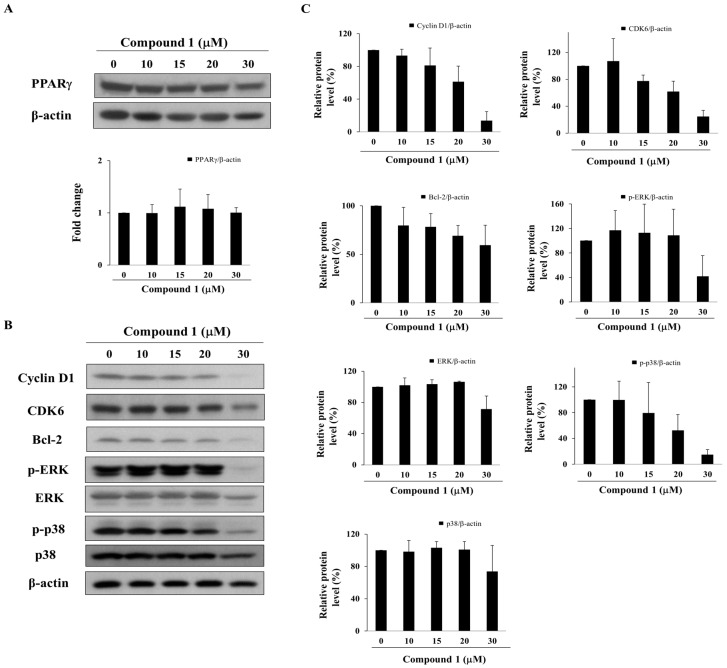
Effect of compound **1** on the expression of PPARγ and PPARγ-target gene products in MCF-7 cells. (**A**) Upper panel, the expression of PPARγ after treatment with compound **1** for 48 h in MCF-7 cells. Lower panel, fold change of PPARγ/β-actin in compound **1**-treated MCF-7 cells compared with DMSO control (*n* = 3); (**B**) Western blot analysis of compound **1** on the phosphorylation and/or expression of cyclin D1, CDK6, Bcl-2, ERK, and p38. MCF-7 cells were exposed to compound **1** at indicated concentrations for 48 h; (**C**) Relative protein expression of cyclin D1/β-actin, CDK6/β-actin, Bcl-2/β-actin, p-ERK/β-actin, ERK/β-actin, p38/β-actin and p-p38/β-actin in compound **1**-treated MCF-7 cells compared with DMSO control (*n* = 3).

Although the expression of PPARγ remained relatively unaltered after treatment with compound **1**, compound **1** at ≥ 20 µM suppressed the expression levels of PPARγ-targeted gene products, including cyclin D1, CDK6 [6], and Bcl-2 [35], which govern cell cycle progression and apoptosis (Figure 4A,B). Moreover, reminiscent of the PPARγ activators pioglitazone and prostaglandin (PG)J2, compound **1** also downregulated the phosphorylation of ERK and p38 (Figure 4B,C), which have been reported to interrupt cell proliferation [36,37].

### 2.5. Compound ***1*** Increases Reactive Oxygen Species (ROS) Generation

**Figure 5:**
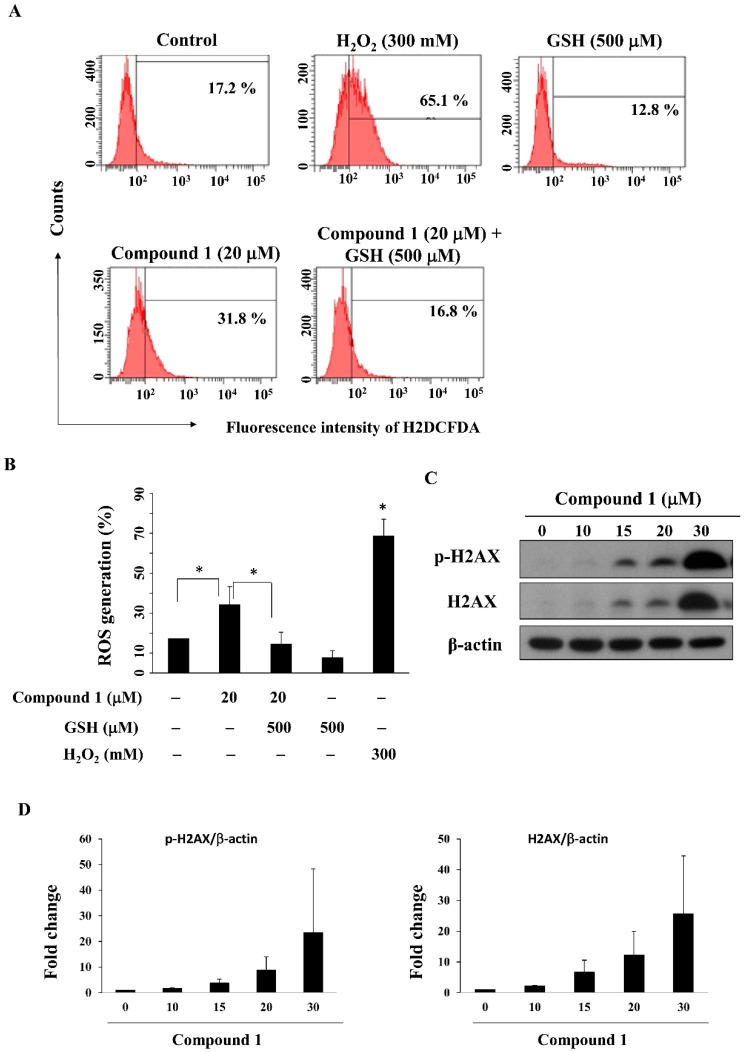
Compound **1** increased ROS generation and DNA damage. (A) Cells were stained with an ROS fluorescence probe, carboxy-DCFDA, after treatment with compound **1** in the presence or absence of glutathione (GSH) for 24 h and examined by flow cytometry; 300 mM H_2_O_2_ was used as the positive control; (**B**) Bar chart representing the results of the flow cytometry analysis of MCF-7 cells stained with carboxy-DCFDA at the indicated treatment doses (*n* = 3). * *p* < 0.05; (**C**) Western blot analysis of the phosphorylation and expression of H2AX after treatment of MCF-7 cells with compound **1** for 48 h; (**D**) Fold changes of p-H2AX/β-actin (left panel) and H2AX/β-actin (right panel) in compound **1**-treated MCF-7 cells as compared to DMSO control (*n* = 3).

PPARγ activators including pioglitazone and rosiglitazone have been reported to inhibit the growth of lung cancer cells by increasing ROS production [38,39]. We examined the ability of compound **1** to increase ROS generation in MCF-7 cells by using flow cytometry analysis. As shown in Figure 5A,B, compound **1** increased ROS generation relative to the control group, from 17.2% to 31.8% after a 24-h treatment, which could be partially reversed by pre-treatment with the antioxidant glutathione (GSH); 300 mM H_2_O_2_ was used as a positive control. As ROS can cause DNA damage response [40], we further examined whether compound **1** causes DNA damage. Our results showed that compound **1** upregulated both p-H2AX and H2AX, both of which are biomarkers of DNA double strand break formation [41], in MCF-7 cells (Figure 5C,D).

### 2.6. Compound ***1*** Induces Autophagy

**Figure 6:**
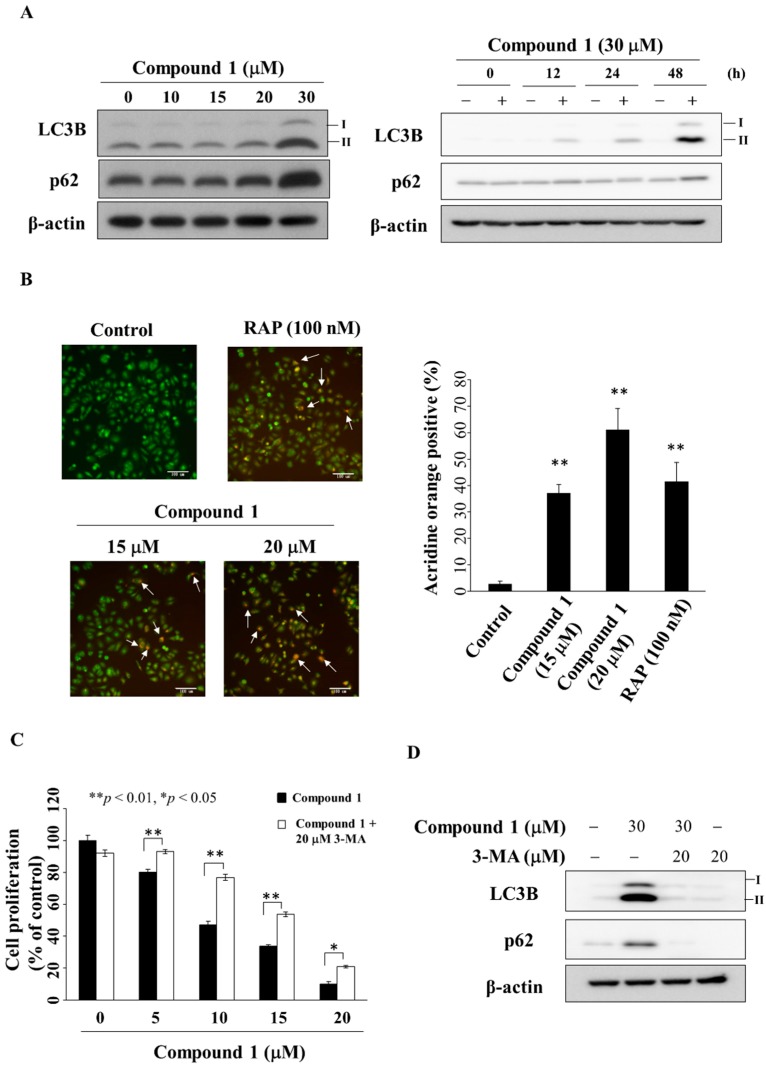
Compound **1** induced autophagy. (**A**) The left panel shows the expression of LC3B-II and p62 in cells treated with compound **1** for 48 h. The right panel shows the expression of LC3B-II and p62 in MCF-7 cells after treatment with compound **1** (at 30 μM) at the times indicated; (**B**) Left, the autophagosomes stained with acridine orange. The white arrows indicate acidic vesicle. Cells were treated with compound **1** or DMSO or 100 nM rapamycin (RAP) for 48 h, and visualized under a fluorescence microscope (200× magnification). The right-hand side shows the percentage of cells stained positively with acridine orange. At least 100 cells from each treatment were examined under fluorescence microscopy. Data are represented as the mean ± S.D. ** *p* < 0.01 compared to control; (**C**) The anti-proliferative effect of compound **1** or in combination with 20 μM 3-methyladenine (3-MA) in MCF-7 cells. Cells were treated with DMSO, with compound **1** alone, or a combination of both for 24 h, and cell proliferation was analyzed by MTT assays. Points, mean; bars, S.D. (*n* = 6). * *p* < 0.05, ** *p* < 0.01; (**D**) Effect of LC3B and p62 after the treatment of compound **1** or 3-MA or in combination with 20 μM 3-MA in MCF-7 cells.

Previous studies have shown that PPARγ activators troglitazone and resveratrol inhibited cell growth through the activation of autophagy in bladder and colon cancer cells [42,43]. Western blotting showed that compound **1** increased the expression of LC3B-II [44] and p62 [44] in a dose- and time-dependent manner in MCF-7 cells (Figure 6A). This compound **1**-induced upregulation of LC3B-II and p62 was also noted in MDA-MB-231 cells (Figure S3). Due to compounds **2** and **3** showing marginal PPARγ activities, we also examined the ability of these two compounds to induce autophagy. As shown in Figure S4, compound **2** and PPARγ activator troglitazone up-regulated the expression of p62 and LC3B-II, which, however, was not observed with compound **3**. In the course of autophagy, formation of acidic vesicular organelles (AVOs) is a characteristic feature of autophagic cells [45]. Our data showed that compound **1** increased the proportion of red-stained AVOs after 24 h in a dose-dependent manner in MCF-7 cells (Figure 6B, rapamycin was used as a positive control). Following treatment with compound **1**, the percentage of AVOs was calculated from the images, which revealed a concentration-dependent increase after 24 h (Figure 6C). To further elucidate the role of autophagy in compound **1**-mediated inhibition of cell proliferation, MCF-7 cells were co-treated with the autophagy inhibitor 3-methyladenine (3-MA) [46]. MTT assays show that 3-MA could partially rescue cells from the suppressive effect of compound **1** on cell proliferation (Figure 6C). Furthermore, Western blot analysis showed the ability of 3-MA (20 µM) to diminish compound **1** (30 µM)-mediated upregulation of the expression of LC3B-II and p62 in MCF-7 cells (Figure 6D).

## 3. Experimental Section

### 3.1. Marine Compound Library

Compounds **1**–**11** were isolated from various marine invertebrates as previously described [19,20,21,22,23,24,25,26,27].

### 3.2. Chemicals and Reagents

All of the chemicals were dissolved in DMSO, and were added to culture medium at indicated concentrations with a final DMSO concentration of less than 0.1%. Rabbit polyclonal antibodies against various biomarkers were obtained from the following sources: cyclin-dependent kinase (CDK)6, LC3B, p-^180/182^Thr/Tyr p38, p38, Bcl-2, p-H2AX, H2AX, p-^202/204^Thr/Tyr ERK, ERK, PARP, p62, caspase-3, PPARγ, cyclin D1 (Cell Signaling Technologies, Beverly, MA, USA), and β-actin (Sigma-Aldrich, St. Louis, MO, USA). The enhanced chemiluminescence (ECL) system for detection of immunoblotted proteins was purchased from GE Healthcare Bioscience (Piscataway, NJ, USA). The peroxisome proliferator-activated receptor response element (PPRE)-x3-TK-Luc plasmid was kindly provided by Dr. Ching S. Chen (China Medical University Hospital). GW9662, 3-methyladenine, rapamycin, and other chemical and biochemical reagents were obtained from Sigma-Aldrich unless otherwise mentioned.

### 3.3. Cell Culture

MCF-7 breast cancer cells were purchased from the American Type Culture Collection (Manassas, VA, USA), and cultured in DMEM/Ham’s F-12 medium (Gibco, Grand Island, NY, USA). These cells were supplemented with 10% heat-inactivated fetal bovine serum (FBS) and antibiotics (10 mg/mL of neomycin, 5 mg/mL of penicillin, and 5 mg/mL streptomycin) at 37 °C in a humidified incubator containing 5% CO_2_.

### 3.4. Cell Viability

The suppressive effects of compounds on cell viability were assessed using the 3-(4,5-dimethylthiazol-2-yl)-2,5-diphenyl-2*H*-tetrazolium bromide (MTT) assay [28]. Briefly, cells were seeded and incubated in 96-well, flat-bottomed plates in 10% FBS-supplemented medium for 24 h and were exposed to various concentrations of compounds dissolved in DMSO (final DMSO concentration, 0.1%) in 5% FBS-supplemented medium. Then, the medium was removed and replaced by 200 μL of 0.5 mM MTT in 10% FBS-containing DMEM/Ham’s F-12 medium, and cells were incubated in the 5% CO_2_ incubator at 37 °C for 3 h. After removing the supernatants, the MTT dye was solubilized in DMSO. Absorbance at 570 nm was determined on a plate reader. The inhibition of cell proliferation was expressed as a percentage of the viable cells of control culture condition. The IC_50_ values of each group were calculated by the median-effect analysis and presented as the mean ± standard deviation (S.D.).

### 3.5. Flow Cytometry Analysis

Cell cycle analysis was performed by flow cytometry [28]. Briefly, cells were plated in 6-well plate and treated with DMSO, or compound **1** or etoposide at the indicated concentration for 48 h with 5% FBS-supplemented DMEM/F12. After collecting the cells, cells were fixed in 70% cold ethanol for 4 h at 4 °C followed by spinning at 1200 rpm for 5 min and re-suspending in ice-cold phosphate-buffered saline (PBS) containing 2% FBS. Then, cells were stained with propidium iodide (PI) and analyzed by flow cytometry (Becton Dickinson, Heidelberg, Germany) and the multicycler (ModFitLT 3.0, Becton Dickinson, Germany) software program. ROS production was detected using the fluorescence probe [5-(and-6)-carboxy-2′,7′-dichlorodihydrofluoresceindiacetate (carboxy-DCFDA)] [47]. Cellular ROS contents were detected by flow cytometry according to the manufacturer’s instruction.

### 3.6. Detection of Autophagosomes by Staining with Acridine Orange (AO)

Cells were plated in 6-well plate and treated with DMSO or drug at the indicated concentration for 24 h as previously described [48]. Then cells were incubated with medium with acridine orange (1 μg/mL) at 37 °C for 15 min, the acridine orange was removed, cells were washed once with PBS, fresh media was added, and examined with under a microscope (Axiovert 200M, Carl Zeiss, Göttingen, Germany).

### 3.7. Western Blot Analysis

After collecting the drug-treated cells and washing with ice-cold PBS, the cells were resuspended in lysis buffer [28]. Soluble cell lysates were collected after centrifugation at 1500 *g* for 5 min. Equivalent amounts of protein (60–100 μg) from each cell lysate were resolved in 10% sodium lauryl sulfate (SDS)-polyacrylamide gels. Bands were transferred to nitrocellulose membranes and blocked with 5% nonfat milk in PBS containing 0.1% Tween 20 (PBST) and incubated overnight with the corresponding primary antibodies (1:1000–1:2000) at 4 °C. After washing with PBST four times, the membrane was incubated with the secondary antibody (1:1000) with PBST at room temperature for 1 h and visualized by the ECL.

### 3.8. Statistical Analysis

Data were presented as means ± S.D. Statistical analysis was performed using the Student’s *t*-test method for two group comparison. Values of *p* < 0.05 were considered statistically significant.

## 4. Conclusions

Marine natural products have served as sources of therapeutic agents over the past three decades [49]. Accumulating evidence indicates that PPARγ is a potential therapeutic target not only for the treatment of type II diabetes and inflammation but also for cancer. Several studies have revealed the activities of marine natural products in inhibiting inflammation in leukemia cells and in inducing apoptosis in breast cancer cells via PPARγ activation [50]. For example, excavatolide B, a diterpene isolated from marine corals, exhibited anti-tumor effects by altering PTEN/Akt and PPARγ signaling pathways in lung cancer cells [51]. In the present study, a bioassay-based screening was used to identify PPARγ activators from an in-house marine compound library. Our results demonstrated that among the 11 compounds, compound **1,** a sterol, showed the ability to activate PPARγ activation, to induce PPARγ-dependent apoptosis and autophagy in MCF-7 cells.

Several downstream effector/mediators of PPARγ have been reported to underlie the antitumor effects of PPARγ activators. For example, troglitazone induced G1 arrest and apoptosis by reducing the expression of cyclin D1 and CDK6 in breast cancer cells [6]. Two PPARγ activators PGJ2 and pioglitazone were reported to induce apoptosis by decreasing Bcl-2 in chronic hepatitis B-associated hepatocellular carcinoma cells [52]. Consistent with these findings, our results showed that compound **1** inhibited the expression of PPARγ-mediated genes including cyclin D1, CDK6, and Bcl-2. Recently, Fujita et al. reported that troglitazone inhibited cell growth by increasing the expression of p-p38 and p-ERK in pancreatic cancer *in vitro* and *in vivo* [32]. However, our study found that compound **1** down-regulated the phosphorylation of p38 and ERK in MCF-7 cells. We rationalize that this discrepancy might be attributable to difference in cell lines used and, equally important, PPARγ-independent antitumor mechanisms that have been reported for troglitazone [53].

Excessive production of ROS has been associated with the development and progression of breast cancer [54]. However, ROS generation also represents a major mechanism by which many chemotherapeutic agents mediate their antitumor effect. For instance, resveratrol sensitized mesothelioma cells to cisplatin, in part, by inducing excessive rise in ROS [55]. The endogenous PPARγ activator 15d-PGJ2 was also reported to induce ROS-mediated JNK activation which contributes to apoptosis in osteosarcoma [56]. In addition, overproduction of ROS is also associated with increases in DNA damage, leading to genetic instability [57]. PPARγ activators have been shown to enhance sensitivity to γ-radiation by inducing γ-H2AX expression and apoptosis [58]. Consistent with these reports, compound **1** increased both phosphorylation and expression of H2AX in MCF-7 cells.

Evidence indicates that autophagy can be a double-edged sword in the context of its role in regulating tumor growth [59,60]. For example, a previous study showed that allelic loss of autophagy gene, Beclin 1, increases the risk of patients developing the aggressive HER2-positive breast cancer [61]. However, LC3B is one of the positive predictors for longer breast cancer survival after adjuvant chemotherapy [62]. In this study, we observed that autophagy was induced following treatment with compound **1**, as evidenced by the upregulation of LC3B-II and p62 as well as by the presence of acidic vesicles in the cytoplasm. Moreover, the addition of autophagy inhibitor 3-MA decreased compound **1**-induced cytotoxicity and the expression of LC3B-II in MCF-7 cells, which suggested that compound **1** induces tumor-suppressive autophagy.

## Supplementary Materials

Effects of compound **1** in MDA-MB-231 cells and the expression of LC3B-II of compounds **2**, **3**, and troglitazone in MCF-7 cells are available online at http://www.mdpi.com/1660-3397/16/7/238/s1, Figure S1: Inhibition effects of compound **1** relative to DMSO control on the cell proliferation in MDA-MB-231 cells, Figure S2: Statistically analysis of cell cycle after the treatment of compound **1** in MDA-MB-231 cells, Figure S3: Expression of p62 and LC3B in compound **1**-treated MDA-MB-231 breast cancer cells for 48 h., Figure S4: Western blot analyses of LC3B in compound **2** or compound **3** or 50 μM troglitazone (TRO) for 48 h in MCF-7 cells.
