# Supplementary material for: A Sterol from Soft Coral Induces Apoptosis and Autophagy in MCF-7 Breast Cancer Cells
Source: Mar Drugs. 2018 Jul 17;16(7):238. doi: 10.3390/md16070238 (PMC6071057; doi:10.3390/md16070238)
Supplement: Supplementary file 1 [file marinedrugs-16-00238-s001.pdf]

Supporting Information

A Sterol from Soft Coral Induces Apoptosis and Autophagy in MCF-7 Breast Cancer Cells

Jing-Ru Weng [1,2], Chang-Fang Chiu [3,4], Jing-Lan Hu [1], Chia-Hsien Feng [5], Chiung-Yao Huang [1], Li-Yuan Bai [3,6,*], and Jyh-Horng Sheu [1,2,7,8,*]

[1] Department of Marine Biotechnology and Resources, National Sun Yat-sen University, Kaohsiung 80424, Taiwan; jrweng@mail.nsysu.edu.tw (J.-R.W.); m055020016@student.nsysu.edu.tw (J.-L.H.); huangcy@mail.nsysu.edu.tw (C.-Y.H.)
[2] Department of Medical Research, China Medical University Hospital, Taichung 40447, Taiwan
[3] Division of Hematology and Oncology, Department of Internal Medicine, China Medical University Hospital, Taichung 40402, Taiwan; d5686@mail.cmuh.org.tw
[4] Cancer Center, China Medical University Hospital, Taichung 40447, Taiwan
[5] Department of Fragrance and Cosmetic Science, College of Pharmacy, Kaohsiung Medical University, Kaohsiung 80708, Taiwan; chfeng@cc.kmu.edu.tw
[6] College of Medicine, China Medical University, Taichung 40402, Taiwan
[7] Graduate Institute of Natural Products, Kaohsiung Medical University, Kaohsiung 80708, Taiwan
[8] Frontier Center for Ocean Science and Technology, National Sun Yat-sen University, Kaohsiung 80424, Taiwan

Figure	Caption	Page
S1	Inhibition effects of compound **1** relative to DMSO control on the cell proliferation in MDA-MB-231 cells. Cells were treated with compound **1** at indicated concentrations for 24 h or 48 h, and cell proliferation was determined by MTT assay. Points, means; bar, S.D. (n = 4-6). ***p* < 0.01.	3
S2	Statistically analysis of cell cycle after the treatment of compound **1** in MDA-MB-231 cells. Cells were treated with compound **1** at the indicated concentrations for 48 h, followed by PI staining and flow cytometric analysis. Treatment with etoposide (ETO) at 10 μM was used as the positive control. Points, mean; bars, S.D. (n = 4). **p* < 0.05, ***p* < 0.01.	4

S3	Expression of p62 and LC3B in compound **1**-treated MDA-MB-231 breast cancer cells for 48 h.	5
S4	Left, Western blot analyses of LC3B in compound **2** or compound **3** or 50 μM troglitazone (TRO) for 48 h in MCF-7 cells. Three independent experiments were performed, and the fold changes of LC3B-II/β-actin is presented in right panel. Points, means; bars, S.D. (n = 3).	6

Supplementary materials and methods

Inhibition effects of compound 1 relative to DMSO control on the cell proliferation in MDA-MB-231 cells.

Cells (5x103) were treated with compound 1 or DMSO in 5% FBS-supplemented DMEM/F12 medium in 96-well plates. After 24 h, the medium was removed, replaced by 200 μL DMEM/F12 containing 0.5 mg/mL of MTT, and cells were incubated in the CO2 incubator at 37°C for 2 h. Supernatants were aspirated from the wells, and the reduced MTT dye was solubilized in 200 μL/well DMSO. Absorbance at 570 nm was measured using a plate reader.

Cell cycle analysis of compound 1 in MDA-MB-231 cells

Cells (2x105/3 mL) were treated with DMSO or compound 1 for 48 h and washed twice with PBS, followed by staining with propidium iodide (PI) and analyzed by flow cytometry and the multicycler (ModFitLT 3.0) software program.

Western blot analysis

Western blot analysis was performed as previously report (Weng et. al, (2017) Phytother. Res., 31, 1722-1730).

Figure S1

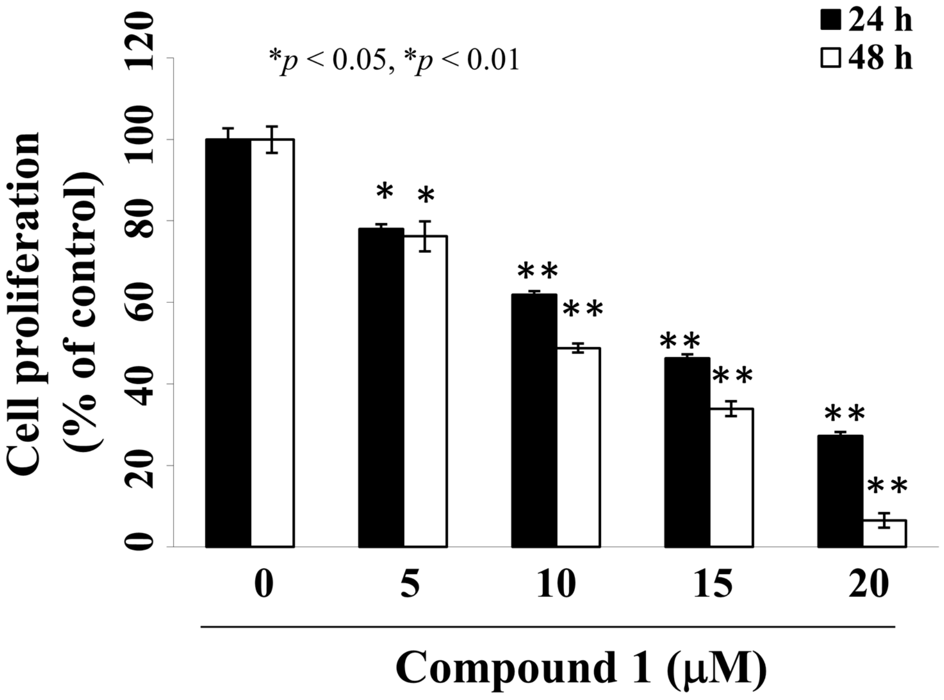
24 h
48 h
*p < 0.05, *p < 0.01
Cell proliferation
(% of control)
0
20
40
60
80
100
120
0
5
10
15
20
Compound 1 (μM)

Figure S2

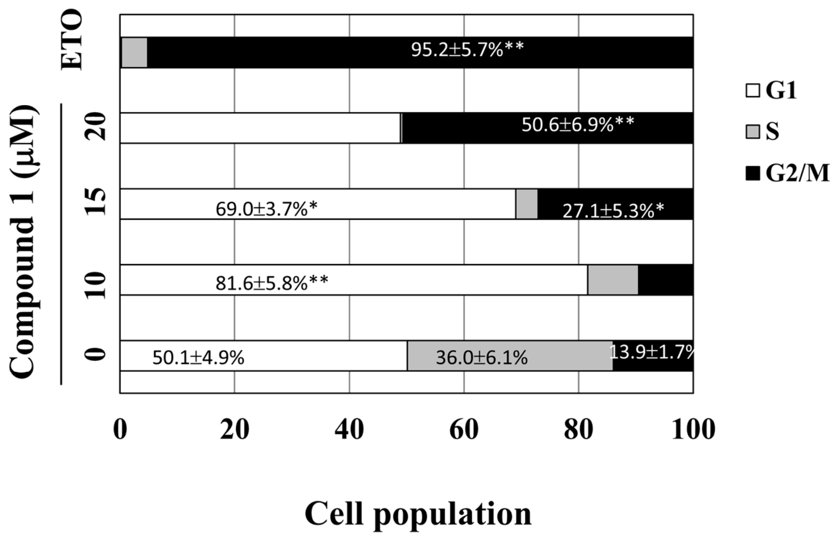
Compound 1 (μM)
ETO
20
15
10
0
95.2±5.7%**
50.6±6.9%**
69.0±3.7%*
27.1±5.3%*
81.6±5.8%**
50.1±4.9%
36.0±6.1%
13.9±1.7%
0
20
40
60
80
100
Cell population
G1
S
G2/M

Figure S3

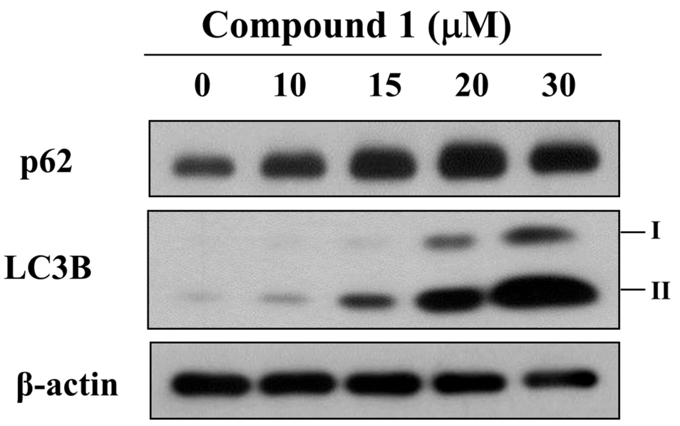

Figure S4

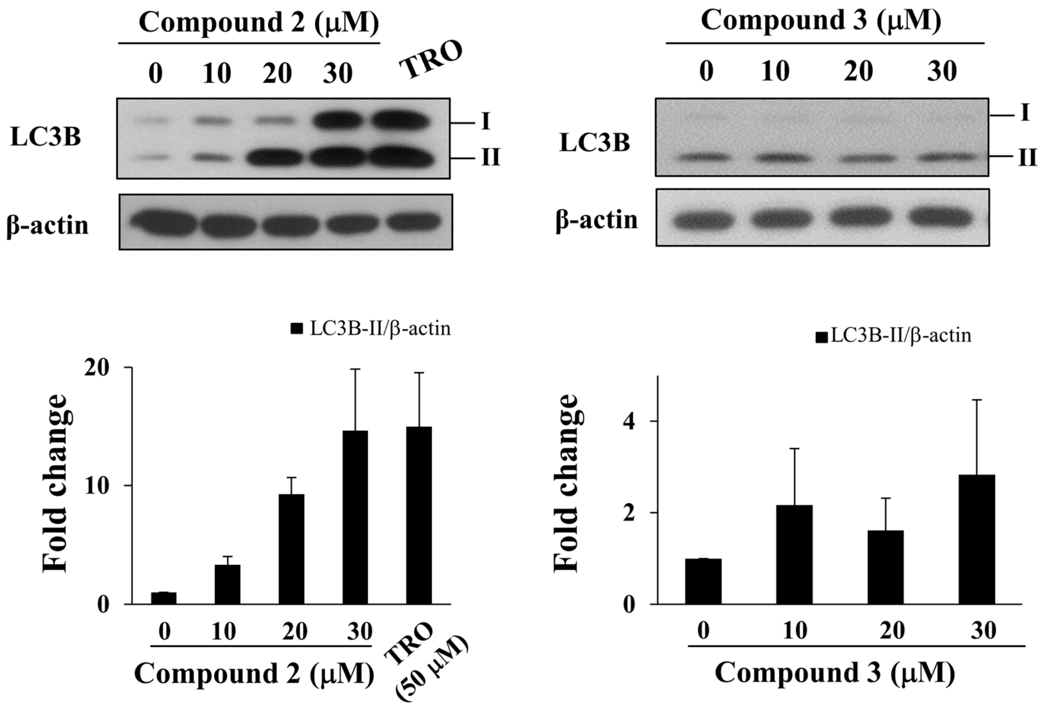
Compound 2 (μM)
0 10 20 30 TRO
LC3B
I
II
β-actin
Compound 3 (μM)
0 10 20 30
LC3B
I
II
β-actin
LC3B-II/β-actin
Fold change
20
10
0
0 10 20 30
TRO (50 μM)
Compound 2 (μM)
LC3B-II/β-actin
Fold change
4
2
0
0 10 20 30
Compound 3 (μM)
